# Highly transparent and flexible Ag nanowire-embedded silk fibroin electrodes for biocompatible flexible and transparent heater†

**DOI:** 10.1039/d0ra05990k

**Published:** 2020-08-28

**Authors:** Jin-Hyeok Park, Hae-Jun Seok, Eswaran Kamaraj, Sanghyuk Park, Han-Ki Kim

**Affiliations:** School of Advanced Materials Science and Engineering, Sungkyunkwan University 2066, Seobu-ro, Jangan-gu Suwon-si Gyeonggi-do 16419 Republic of Korea hankikim@skku.edu; Department of Chemistry, Kongju National University 56, Gongjudaehak-ro Gongju-si Chungcheongnam-do 32588 Republic of Korea spark0920@kongju.ac.kr

## Abstract

We investigated the electrical, optical and mechanical properties of silver (Ag) nanowire (NW) embedded into a silk fibroin (SF) substrate to create high performance, flexible, transparent, biocompatible, and biodegradable heaters for use in wearable electronics. The Ag NW-embedded SF showed a low sheet resistance of 15 Ω sq^−1^, high optical transmittance of 85.1%, and a small inner/outer critical bending radius of 1 mm. In addition, the Ag NW-embedded SF showed a constant resistance change during repeated bending, folding, and rolling because the connectivity of the Ag NW embedded into the SF substrate was well maintained. Furthermore, the biocompatible and biodegradable Ag NW-embedded SF substrate served as a flexible interconnector for wearable electronics. The high performance of the transparent and flexible heater demonstrated that an Ag NW-embedded SF-based heater can act as a biocompatible and biodegradable substrate for wearable heaters for the human body.

## Abstract 2

We demonstrated the characteristics of a transparent, flexible silver nanowire-embedded silk fibroin substrate that can be used as a flexible and biocompatible electrode for wearable electronics.

## Graphical abstract

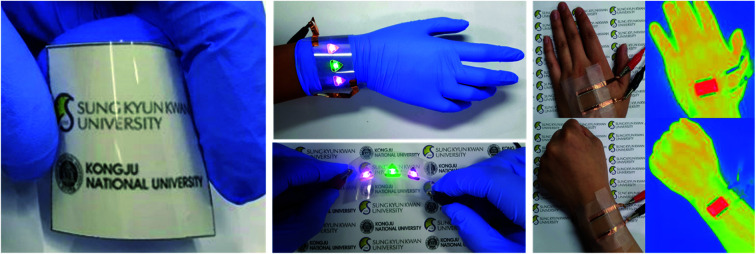

## 1 Introduction

Transparent, flexible, biocompatible and biodegradable electrodes have attracted much interest as key components of electronic skins, biosensors, and implantable defibrillators.^1–7^ During the past few decades, efforts to develop transparent electrode materials have mainly focused on transparent conductive oxide (TCO), metallic nanowires, conducting polymers, and carbon-based electrodes including carbon nanotubes, graphene, and graphene oxides.^8–12^ Although several different kinds of transparent electrode materials have been suggested, Sn-doped In_2_O_3_ (ITO) film coated on a flexible substrate or a glass substrate is most often used in flat panel displays, photovoltaics, touch screen panels, and sensors due to its high conductivity and transmittance. However, normal ITO films on flexible substrates cannot be applied to wearable electronics because ITO film is brittle and typical flexible substrates such as polyethylene (PET) and polyimide (PI) are not biocompatible with the human body.^13^ In addition, the world’s indium resources are restricted because indium makes up only 0.21 parts per million (ppm) of the Earth’s crust and the price of indium has increased in the past decade.^14–19^ Also, the vacuum-based sputtering process used to create ITO film prevents the fabrication of cost-effective electrodes for wearable devices. Compared to commonly used ITO films, Ag nanowire (NW) percolating electrodes exhibit higher conductivity, comparable optical transparency, superior flexibility, and superior biocompatibility.^20–22^ Also, the solution-based printing process used to make Ag NW electrodes is cost-effective and simple. Due to the merits of Ag NWs as flexible electrodes, research groups have reported various applications. Li *et al.* developed physical and chemical sensors and micro-electro-mechanical system (MEMS) devices with Ag NW electrodes.^23^ Also, Gupta *et al.* manufactured antenna devices for radio-frequency identification (RFID) tags.^24^ Our Ag NW electrodes are different in terms of improved flexibility by incorporating the Ag NWs into a silk fibroin (SF) matrix. As a promising biocompatible and biodegradable substrate for Ag NW coating, SF, which is a natural protein fibre obtained from the cocoons of mulberry silkworm larvae, has been extensively reported.^25–27^ Because natural silk is composed of SF coated with silk sericin proteins, the removal of sericin protein enables the manipulation of transparent SF into various forms, such as fibres, gels, films, and foams.^28–36^ Recently, SF has been employed in bioelectronics due to its remarkable characteristics, such as high transmission, favourable mechanical properties, non-toxicity, and biocompatibility. SF substrates show high transmittance of 90–95% at visible wavelengths. Furthermore, SF is obtained by a straightforward dialysis process from the *Bombyx mori* cocoon in high yields.^37–39^ Liu *et al.* reported that Ag NW/SF is a promising conductive and transparent substrate for flexible organic light emitting diodes and can be used as a substitute for typical brittle ITO electrodes.^40^ Min *et al.* demonstrated that patterned Ag NW on a SF substrate acts as a transparent resistor and a radio-frequency antenna for food sensors.^41^ Qi *et al.* reported that Ag NW and SF composite films have excellent transmittance and conductivity and can be used as flexible interconnectors. Although several applications of Ag NW–SF compositions or Ag NW-coated SF substrates, such as bacteria sensors, photonics, electronic devices, food sensors, actuators, therapeutics, and energy harvesting devices have been suggested, their use as electrodes for wearable transparent and flexible heaters (TFHs) has not yet been reported. Generally, TFHs act as heating sources for automobiles, sensors, reaction cells, microchips, and vinyl greenhouses. Notably, TFHs can be attached to the human body as wearable heat sources. Therefore, electrodes with biocompatible and flexible characteristics are necessary for the creation of wearable TFHs.^42–46^

In this work, we investigated the electrical, optical, and mechanical properties of an Ag NW-embedded SF substrate for use as an electrode for wearable and biocompatible TFHs. The mechanical flexibility of the Ag NW-embedded SF substrate was investigated in detail using lab-designed inner/outer bending, folding, and rolling test equipment. By employing the Ag NW-embedded SF substrate to fabricate flexible interconnectors and TFHs, we demonstrate the feasibility of biocompatible Ag NW-embedded SF substrates for wearable electronics and the possibility that they can be substituted for conventional high-cost ITO films.

## 2 Experimental

### 2.1 Preparation of Ag NWs/SF films

**Fig. 1:**
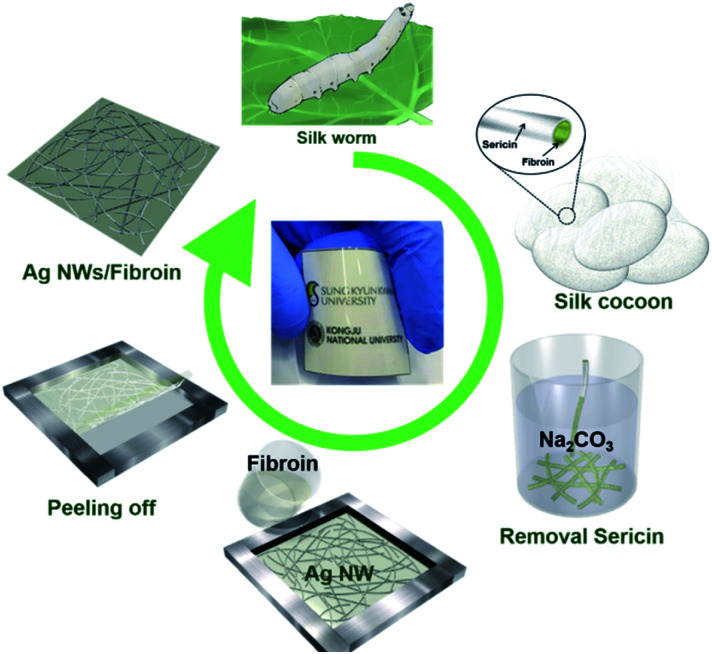
Schematic fabrication procedure of an Ag NW-embedded SF substrate with high transparency and flexibility.

Fig. 1 shows a schematic of the overall fabrication procedure used to produce the Ag NW-embedded SF substrate. *Bombyx mori* silk cocoons were degummed to remove the sericin protein by boiling in 0.02 M Na_2_CO_3_ aqueous solution for 30 min, and rinsed three times with deionized (DI) water.^47^ After squeezing out excess water and drying in an oven at 60 °C for 12 h, the remaining degummed fibroin fibres were dissolved in 9.3 M LiBr solution to obtain fibroin aqueous solution. Then, the solution was dialyzed against DI water using a dialysis tubing cellulose membrane (MW cutoff: 14 000 Da) for 72 h to remove LiBr. Then, the obtained aqueous fibroin solution was centrifuged three times at 9000 rpm for 15 min and stored at 4 °C. To cast an Ag NW-embedded SF substrate for a transparent electrode, Ag NWs were synthesized by a modified polyol process. Briefly, 35 g of polyvinylpyrrolidone (PVP, average mol wt. 40 000) and 80 mg of ZnCl_2_ were dissolved in 300 mL of ethylene glycol at 175 °C with gentle agitation. Then, 100 mL of 0.2 mol L^−1^ AgNO_3_ solution in ethylene glycol was injected dropwise using a syringe into the solution at a rate of 1 mL min^−1^. The solution was heated at 65 °C for 30 min then cooled to room temperature. The synthesized Ag NWs were successively purified with acetone and methanol and centrifuged at 3000 rpm for 10 min to remove the ethylene glycol and excess PVP. Finally, the synthesized Ag NWs (21 nm average diameter, 22 μm average length) were dispersed in isopropanol (0.5 wt%), and bar-coated on poly-ethylene terephthalate (PET) film (Higashiyama) and dried at 85 °C for 10 min. After fabrication of the Ag NW network electrode on PET, aqueous SF solution (5 wt%) was poured to cast a thick film on the prepared Ag NW network electrode. Then, water was removed at 60 °C for 12 h, and the Ag NW-embedded SF substrate was carefully peeled off from the PET substrate to obtain the Ag NWs–SF composite, as illustrated in Fig. 1.

### 2.2 Characterization of the Ag NW/SF substrate

The electrical properties of the Ag NW-embedded SF substrate and reference Ag NWs/PET were measured with Hall effect measurement (HMS-4500, Ecopia). The optical transmittance of the Ag NW-embedded SF substrate was compared with Ag NWs/PET at wavelengths from 350 nm to 1200 nm using a UV/Vis spectrophotometer (V-670, Jasco). The mechanical flexibility of the Ag NW-embedded SF substrate was investigated with a laboratory-created inner/outer bending, rolling, and folding test machine. To determine the critical bending radius of the Ag NW-embedded SF substrate, the resistance change was measured with decreasing bending radius in inner and outer bending tests. Furthermore, dynamic fatigue tests of the Ag NW-embedded SF substrate at a fixed inner and outer bending radius of 3 mm were carried out for 10 000 cycles. Additionally, various types of dynamic flexibility tests, such as rolling and folding tests, were conducted. For the dynamic rolling test, one side of a rectangular sample was anchored at a terminal jig. The other side of the sample was rolled by a rotating cylindrical bar with a radius of 3 mm for 10 000 cycles. In the dynamic folding test, one side of a sample was anchored at a terminal jig and the other side of the sample was folded 10 000 times at a folding angle of 180°. Surface images of the Ag NW-embedded SF substrate before and after the bending test were analysed by field emission-scanning electron microscopy (FE-SEM) (JSM-7600F, JEOL).

### 2.3 Fabrication and evaluation of the thin-film heaters

To demonstrate the feasibility of the Ag NW-embedded SF substrate, flexible interconnectors and TFH were fabricated. A rectangular (15 × 80 mm^2^) interconnector was cut off from the as-fabricated sample using scissors. Commercial light-emitting diode (LED) chips were directly connected to the Ag NW-embedded SF substrate strip. Then, a low voltage of 3 V was applied to the two ends of the flexible Ag NW-embedded SF interconnector. The state of the LEDs was observed during bending and attachment to the human body. In addition, we fabricated TFHs on the Ag NW-embedded SF substrate with a size of 25 × 25 mm^2^. To apply direct current (DC) power to the TFHs, Ag contact electrodes were sputtered on the edge of the Ag NW-embedded SF substrate under a pure Ar atmosphere. DC voltage was supplied by a power supply (OPS 3010, ODA technologies) to the TFHs through an Ag contact electrode at the edge of the film. The temperature of the TFHs was measured using a thermocouple mounted on the surface of the TFHs and an infrared (IR) thermal imager (A35sc, FLIR). The feasibility of the TFH with the Ag NW-embedded SF substrate was also confirmed by attaching the heater to the human body.

## 3 Results and discussion

### 3.1 Electrical, optical, and surface properties of Ag NWs/SF films

**Fig. 2:**
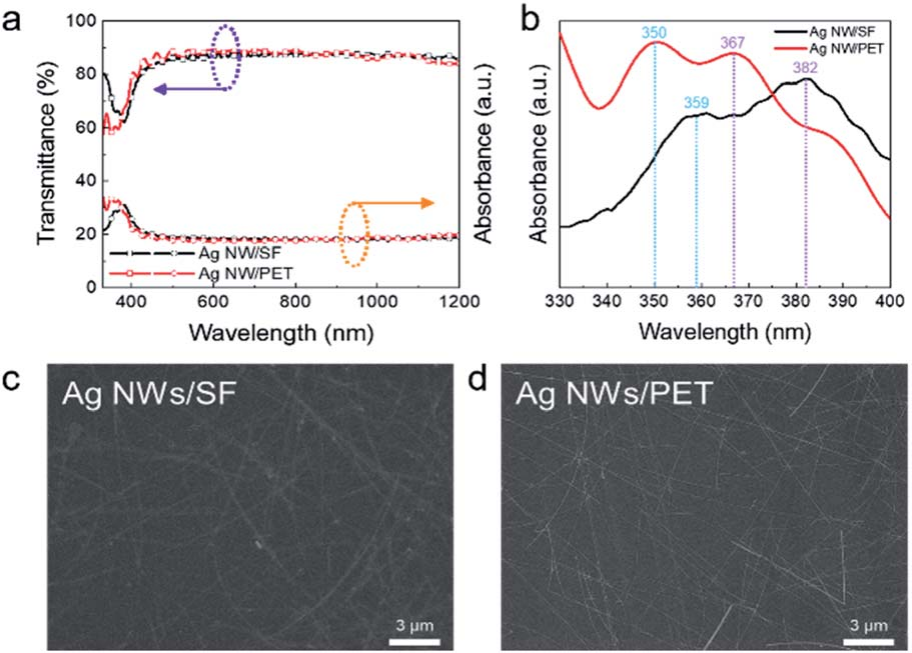
(a) Optical transmittance and (b) absorbance of an Ag NW-embedded SF and Ag NW-coated PET substrate. Comparison of FE-SEM surface images of (c) Ag NW-embedded SF and (d) Ag NWs/PET films.

Fig. 2(a) shows optical transmittance of the Ag NW-embedded SF substrate and Ag NWs/PET films. The Ag NW-embedded SF substrate showed slightly lower optical transmittance due to the high density of the Ag NW network. However, the Ag NW structure that was embedded into the SF substrate showed higher optical transmittance than a reference sample in the short wavelength region below 375 nm. Fig. 2(b) represents the optical absorbance of an Ag NW-embedded SF substrate and Ag NWs coated on a PET substrate. Absorption peaks around 350 nm and 370–380 nm, which were induced by surface plasmon oscillation in Ag NWs, were clearly observed.^48^ The absorption peaks suggest that our synthesized Ag NWs functioned well. Although the optical transmittance of the Ag NW-embedded SF at 550 nm was lower than that of the reference sample, the sheet resistance of the Ag NW-embedded SF substrate had a lower value, as summarized in Table 1. The electrical and optical properties of Ag NW-embedded SF samples prepared under various conditions are summarized in the ESI (Table S1 and Fig. S1†).

**Table 1:** Comparison of sheet resistance and optical transmittance measured in air background of Ag NW electrodes on SF and PET substrates

Material	Sheet resistance (Ω sq^−1^)	Transmittance_550 nm_ (%)
Ag NWs/SF	15	85.1
Ag NWs/PET	20	91.2

Fig. 2(c) and (d) show the surface FE-SEM images of Ag NW-embedded SF and Ag NWs/PET films, respectively. Both Ag NWs showed well-connected network structures regardless of the presence of a flexible substrate. The connectivity of the Ag NWs network allows for the high conductivity of both types of film. However, compared to the Ag NW-embedded SF sample, the Ag NW-coated PET sample showed a clearer Ag NW network image because Ag NWs and SF were mixed on the surface in the Ag NW-embedded SF substrate. However, the good connections of the Ag NWs in the SF matrix could be related to the low sheet resistance of the electrode. In addition, considering the adhesion of the Ag NW network, the embedded Ag NW structure is favourable for the creation of a mechanically flexible Ag NW network compared to Ag NWs just coated on a PET substrate.

### 3.2 Mechanical flexibility of Ag NWs/SF films

**Fig. 3:**
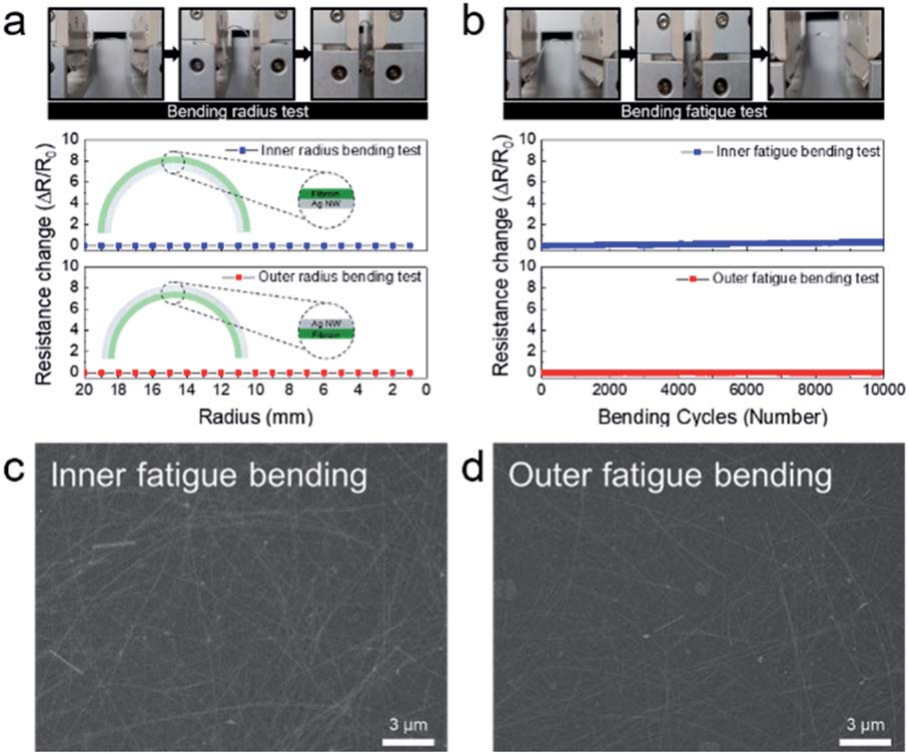
(a) Inner and outer bending test of an optimized Ag NW-embedded SF substrate with decreasing bending radius. (b) Repeated inner and outer bending test results at a fixed bending radius of 3 mm (10 000 trials). Upper panels demonstrate the bending steps in inner/outer bending and fatigue tests. FE-SEM surface images of Ag NWs/SF films after 10 000 cycles of the (c) inner fatigue bending test and (d) outer fatigue bending test.

The mechanical flexibility of the Ag NW-embedded SF substrate was investigated using laboratory-designed inner/outer bending, rolling, and folding tests (Fig. S2†). Fig. 3(a) shows resistance changes of the Ag NW-embedded SF substrate during the inner and outer bending tests with decreasing bending radius. The upper panels depict the outer bending steps. By controlling the gap distance between the electrode which held the sample, the bending radius could be precisely controlled down to 1 mm. The resistance change (Δ*R*) was defined as Δ*R* = (*R* − *R*_0_)/*R*_0_, where *R*_0_ is the initial measured resistance and *R* is the *in situ* measured resistance during the inner/outer radius test. Due to the outstanding mechanical flexibility of the Ag NWs network, there was no resistance change during the inner and outer bending test, even at a bending radius of 1 mm. In addition, as the Ag NWs were uniformly embedded in the SF substrate, the well-connected Ag NWs maintained a network structure even at a small bending radius of 1 mm. In the different bending modes illustrated in the inset, the sample showed no resistance change. Fig. 3(b) shows the dynamic inner and outer bending fatigue tests, such as bending and spreading at a fixed bending radius of 3 mm. Both dynamic inner and outer bending tests of the Ag NW-embedded SF show no resistance change even after 10 000 bending cycles, demonstrating the exceptional flexibility and mechanical stability of the Ag NW-embedded SF sample. Fig. 3(c) and (d) show surface FE-SEM images of the Ag NW-embedded SF substrate after 10 000 cycles. There were no cracks or delamination of the Ag NWs even after 10 000 cycles of inner and outer bending. Each Ag NW-embedded SF substrate still maintained its well-connected network and looked similar to the pristine sample in Fig. 2(c).

**Fig. 4:**
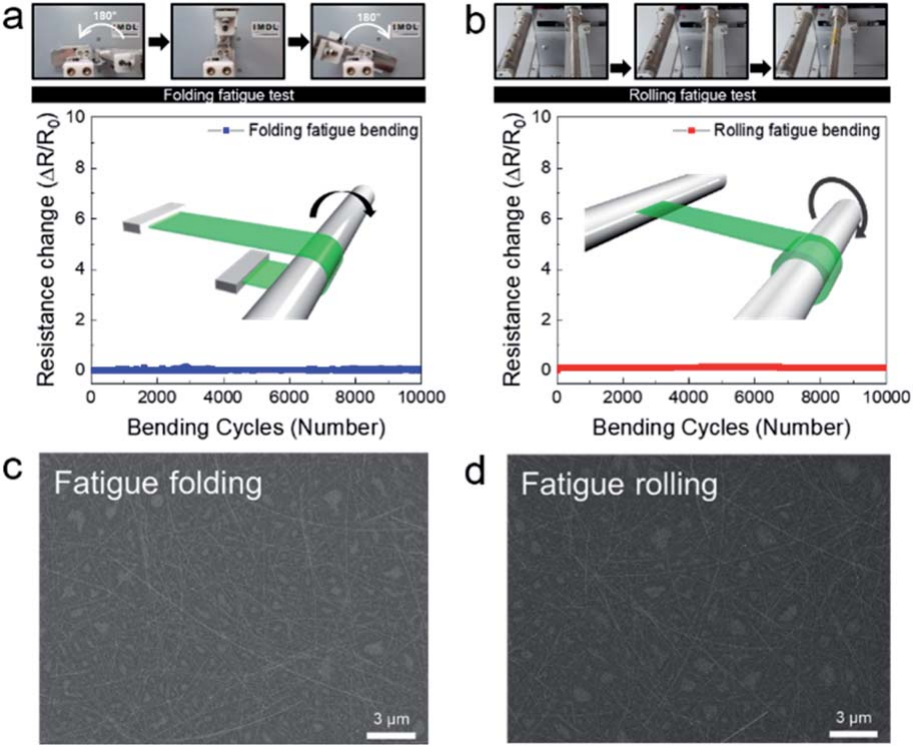
(a) Folding and (b) rolling fatigue test results of the Ag NW-embedded SF sample. Upper panels show the folding and rolling steps. Insets show schematics of the folding and rolling tests. The Ag NW/SF sample was wrapped onto a cylindrical bar. Surface FE-SEM images of the Ag NW/SF substrates after 10 000 cycles of (c) fatigue folding and (d) fatigue rolling tests.

Fig. 4(a) and (b) show the fatigue folding and rolling tests, respectively, of the Ag NW-embedded SF substrate as a function of folding and rolling cycles. The upper panels in Fig. 4(a) and (b) are pictures of the folding and rolling test steps, respectively. The insets in Fig. 4(a) and (b) demonstrate how the Ag NW-embedded SF substrate was folded or rolled on a cylindrical bar with a radius of 3 mm. The sample shows constant resistance during repeated folding and rolling tests. The resistance of the Ag NW-embedded SF substrate was maintained even after 10 000 trials of the folding and rolling tests. Fig. 4(c) and (d) show the surface FE-SEM images of the Ag NW-embedded SF films after 10 000 cycles of the folding and rolling tests, respectively. Even after 10 000 cycles, the surface of the Ag NW-embedded SF substrate appeared to be identical to that of a pristine sample, without cracks or delamination. This also indicates the outstanding flexibility of the Ag NW-embedded SF substrate. The mechanical bending test results demonstrated that Ag NW-embedded SF substrates could be applied as flexible interconnectors in wearable electronics, in which electrodes experience severe stress in various bending modes.

### 3.3 Applications of Ag NW-embedded SF substrates for LED interconnectors and biocompatible thin film heaters

**Fig. 5:**
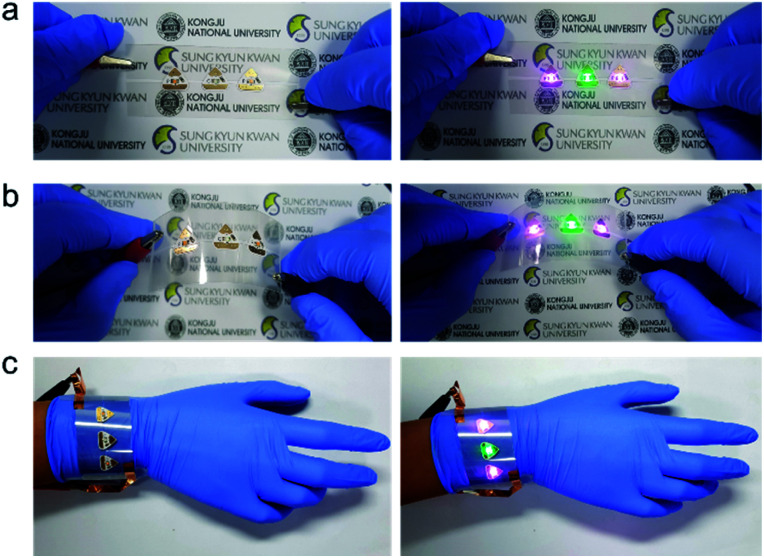
Photographs of an LED interconnector based on Ag NW/SF electrodes. LEDs turned off and on after contacting the Ag NW/SF substrate in the (a) spreading and (b) curved state. (c) Photographs of Ag NW/SF-based interconnector wrapped around a human wrist.

The pictures in Fig. 5 show the Ag NW-embedded SF substrate applied as a flexible interconnector for commercial LEDs. Two sides of the LEDs were directly connected to the Ag NW-embedded SF substrate. AC power was supplied to turn on the LEDs through the flexible Ag NW-embedded SF substrate. Fig. 5(a) and (b) show pictures of LEDs in the off and on states directly connected to the Ag NW-embedded SF substrate. Due to the high conductivity and good flexibility of the Ag NW network, the LEDs remain continuously lit even after curving of the substrate. Our Ag NW-embedded SF can be applied as a wearable interconnector, as shown in Fig. 5(c). The successful operation of the LEDs connected to the Ag NW-embedded SF substrate demonstrates that the Ag NW-embedded SF substrate is promising as an interconnector for wearable electronics.

**Fig. 6:**
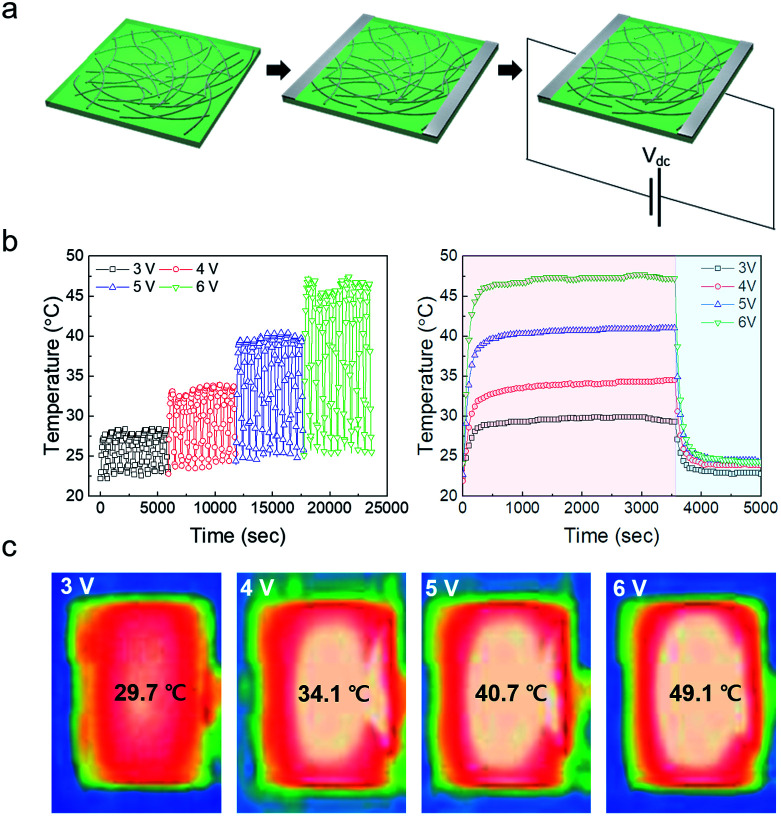
(a) Schematic fabrication process for transparent and flexible TFHs based on Ag NWs/SF electrodes. (b) Temperature profile of Ag NWs/SF-based TFHs as a function of applied voltage for 10 cycles of heating and cooling. (c) Long-term thermal stability test of Ag NWs/SF-based TFHs as a function of applied voltage. (c) IR images of Ag NWs/SF-based TFHs as a function of applied voltage.

Ag NW-embedded SF based TFHs 25 × 25 mm^2^ in size were fabricated to investigate their feasibility as flexible and transparent electrodes. We prepared typical TFHs using a two-terminal metal contact configuration. The fabrication process is illustrated in Fig. 6(a). The two-terminal contact electrodes were 20 nm-thick Ag film deposited by DC sputtering. Fig. S3† shows the detailed fabrication process and structure of the Ag NW-embedded SF-based TFHs. As shown in Fig. S4,† the TFH is transparent enough to show the SKKU logo. Contact metal electrodes were connected to the two terminals of a DC voltage supply. DC voltage was applied to the Ag NWs/SF through sputtered Ag contact electrodes at the edge of the film. The temperature of the Ag NWs/SF-based TFHs was measured by a thermocouple mounted on the surface of the TFHs. Fig. 6(b) shows the temperature profiles of the Ag NWs/SF-based TFHs, plotted with respect to different input DC voltages for 10 cycles of heating and cooling. When DC voltage was applied to the Ag NWs/SF-based TFHs, the temperature of the TFHs gradually increased and reached saturation temperature; it then cooled to the relaxation temperature after the applied voltage was turned off. Even when the relaxation temperature slightly increased during the cyclic thermal heating and cooling tests, the Ag NWs/SF-based TFHs clearly showed repeated operation. The Ag NWs/SF-based TFHs reached a saturation temperature of about 50 °C, which is sufficient for a heat-assisted medical patch, when a DC voltage of 6 V was supplied. Fig. 6(c) shows the temperature profiles of the Ag NWs/SF-based TFHs measured for 1 hour at the respective applied voltages. As shown in Fig. 6(c), the Ag NWs/SF-based TFHs were sustained at saturation temperature for 1 hour and cooled rapidly when the applied voltage disappeared. The results above indicate that our Ag NWs/SF-based electrodes are metallic conductors. Fig. S5† shows the *I*–*V* characteristics of Ag NWs/SF-based electrodes measured *in situ* with temperature profiles. The *I*–*V* characteristics of Ag NWs/SF electrodes showed ohmic behavior (*V* = *IR*). Fig. 6(d) shows the *IR* thermal images of Ag NWs/SF-based TFHs as a function of applied voltage. The temperature is distributed uniformly between the contact terminals. The principle of heating in Ag NWs/SF-based TFTs is Joule’s law, as expressed in the following equation.^49^

1 *V*^2^/*R* = *mC*d*T*(*t*)/d*t* + *A*(*h*_1_ + *h*_2_)(*T*(*t*) − *T*_0_)+*σA*(*ε*_1_ + *ε*_2_)(*T*(*t*)^4^ − *T*_0_^4^)

Here, *V*^2^/*R* is the power input to the TFHs, *V* is the input DC voltage between contact electrodes and *R* is the resistance of the TFHs. *mC*d*T*(*t*)/d*t* is the loss due to conduction in the substrate of mass *m* and specific heat *C*. *A*(*h*_1_ + *h*_2_)(*T*(*t*) − *T*_0_) is heat loss by convection in air, where *A* and *h* are the area of the sample and convective heat-transfer coefficient, respectively. *σA*(*ε*_1_ + *ε*_2_)(*T*(*t*)^4^ − *T*_0_^4^) is heat loss due to radiation from flexible TFHs, where *ε* is the emissivity of the sample involved in the Ag NWs and the SF substrates. The radiation term can be disregarded at low temperatures. Eqn (1) can be expressed in terms of *T*:2
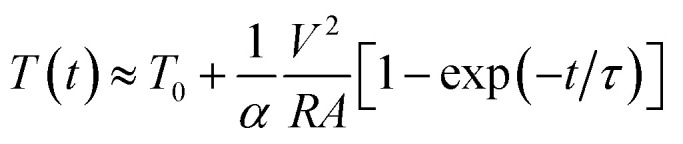
Here, *α* is the transfer constant, which is related to the time constant *σ*, and *τ* is *αA*/*mC*. In eqn (2), the input DC voltage (*V*) and the resistance of the TFHs (*R*) are the only variable values and the others are constant in a given sample. Therefore, the above equation expresses that the lower sheet resistance of TFHs causes a higher saturation temperature at constant voltage.

**Fig. 7:**
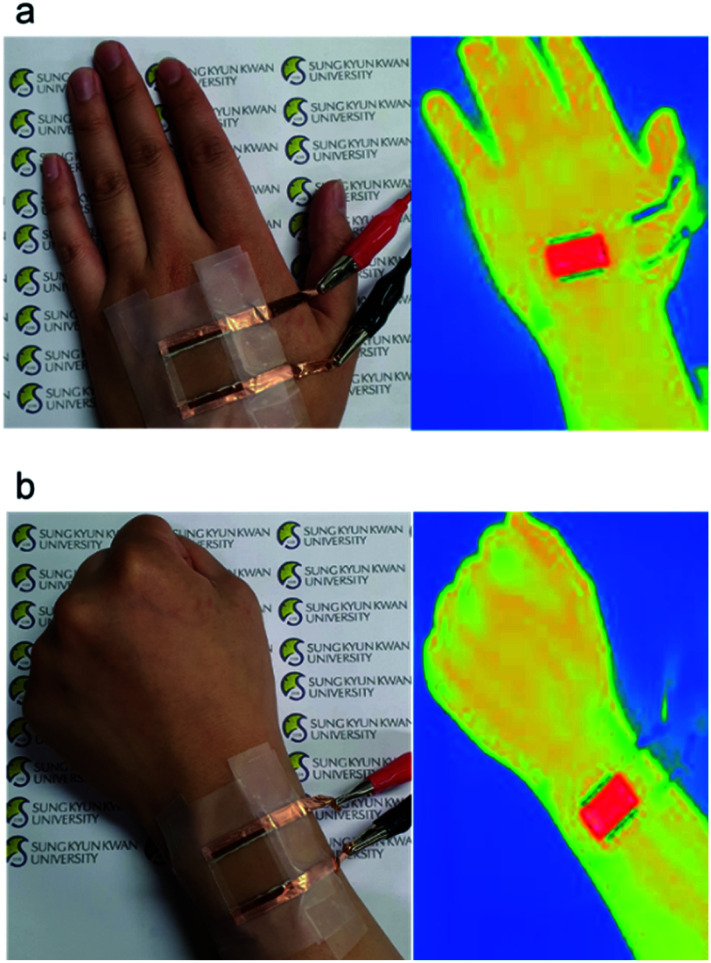
Photographs and IR images of Ag NWs/SF-based TFHs attached to a human (a) hand and (b) wrist.

Several applications can be imagined given the novel properties of Ag NWs and SF in the field of flexible health care; their high transparency and biocompatibility are of particular interest. In addition, Ag NWs and SF can be produced easily and formed into film by a simple solution process. Fig. 7 shows Ag NWs/SF film-based flexible and wearable TFHs attached to a human hand and wrist. When 6 V was applied to the TFHs in Fig. 7, the temperature of the TFHs increased to 45 °C, which is sufficient to utilize them as a source of heat in wearable biological and/or medical devices.

**Fig. 8:**
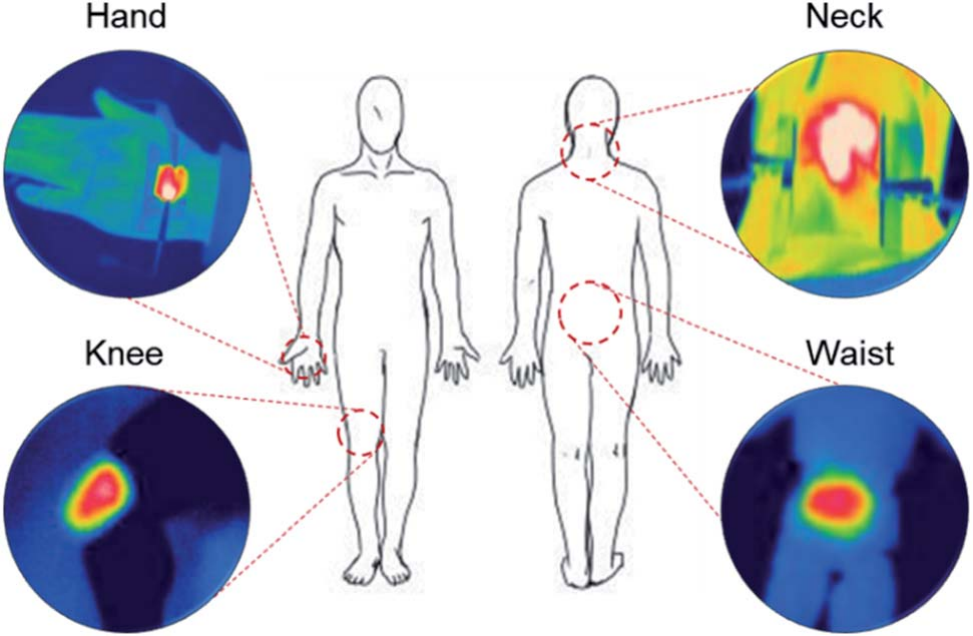
Illustration of the human body and IR images of Ag NWs/SF film-based TFHs attached to the hand, neck, knee, and waist.

Fig. 8 shows IR images of Ag NW-embedded SF-based TFHs attached to the human body, in particular the hand, neck, knee, and waist. Due to the superior flexibility of Ag NW-embedded SF films, the Ag NW-embedded SF-based TFHs attached well to the human body, even to curvy body parts. We anticipate that Ag NW-embedded SF-based TFHs can be used as a heat source in bio-patches due to their high transparency and flexibility. The heat generated from Ag NW-embedded SF-based TFHs can be used to accelerate the absorption of medicine in medical patches, as shown in Fig. 8.

## 4 Conclusions

In summary, we demonstrated the characteristics of a transparent, flexible Ag NW-embedded SF substrate that can be used as a flexible and biocompatible electrode for wearable electronics. The Ag NW-embedded SF substrate prepared by a solution process showed a low sheet resistance of 15 ohms per square, optical transmittance of 85.1%, and critical bending radius of 1 mm, which makes it acceptable for use as an electrode for wearable interconnectors and TFHs. In addition, the Ag NW-embedded SF substrate showed no resistance change and no surface cracks during 10 000 cycles of fatigue bending, folding, and rolling tests. The mechanical test results indicate the superior flexibility of the Ag NW-embedded SF substrate, which can thus be used to fabricate high-performance flexible TFHs. The time–temperature profiles of the TFHs with the Ag NW-embedded SF substrate exhibit a rapid saturation time even at a low voltage due to the low sheet resistance of the Ag NW network. The heat generating, biocompatible Ag NW-embedded SF substrate will have new applications in bioelectronics and medical devices, such as medical patches.

## Conflicts of interest

There are no conflicts to declare.

## Supplementary Material

RA-010-D0RA05990K-s001

## References

[cit1] Kim Y., Kim J.-W. (2016). Appl. Surf. Sci..

[cit2] Horiuchi H., Agetsuma M., Ishida J., Nakamura Y., Cheung D. L., Nanasaki S., Kimura Y., Iwata T., Takahashi K., Sawada K., Nabekura J. (2020). Nat. Commun..

[cit3] Young D., Cong P. (2019). Sens. Actuators, A.

[cit4] Zeng X., Wang Z., Zhang H., Yang W., Xiang L., Zhao Z., Peng L.-M., Hu Y. (2019). ACS Appl. Mater. Interfaces.

[cit5] Bhat K. S., Ahmad R., Yoo J.-Y., Hahn Y.-B. (2018). J. Colloid Interface Sci..

[cit6] He L., Liu Q., Zhang S., Zhang X., Gong C., Shu H., Wang G., Liu H., Wen S., Zhang B. (2018). Electrochem. Commun..

[cit7] Yoon S., Kim H.-K. (2020). Surf. Coat. Technol..

[cit8] Niemelä J.-P., Macco B., Barraud L., Descoeudres A., Badel N., Despeisse M., Christmann G., Nicolay S., Ballif C., Kessels W. M. M., Creatore M. (2019). Sol. Energy Mater. Sol. Cells.

[cit9] Lee J.-E., Kim H.-K. (2019). Sci. Rep..

[cit10] Wu P., Cheng S., Yao M., Yang L., Zhu Y., Liu P., Xing O., Zhou J., Wang M., Luo H., Liu M. (2017). Adv. Funct. Mater..

[cit11] Høiaas I. M., Mulyo A. L., Vullum P. E., Kim D.-C., Ahtapodov L., Fimland D.-O., Kishino K., Weman H. (2019). Nano Lett..

[cit12] Sa K., Mahanandia P. (2019). Thin Solid Films.

[cit13] Pandey M., Wang Z., Kapil G., Baranwal A. K., Hirotani D., Hamada K., Hayase S. (2019). Adv. Eng. Mater..

[cit14] Langley D., Giusti G., Mayousse C., Celle C., Bellet D., Simonato J.-P. (2013). Nanotechnology.

[cit15] He W., Ye C. (2015). J. Mater. Sci. Technol..

[cit16] Zilberberg K., Riedl T. (2016). J. Mater. Chem. A.

[cit17] Rao K. D. M., Kulkarni G. U. (2014). Nanoscale.

[cit18] Kim T. Y., Kim Y. W., Lee H. S., Kim H., Yang W. S., Suh K. S. (2013). Adv. Funct. Mater..

[cit19] Qi N., Zhao B., Wang S.-D., Al-Deyab S. S., Zhang K. Q. (2015). RSC Adv..

[cit20] Sannicolo T., Lagrange M., Cabos A., Celle C., Simonato J.-P., Bellet D. (2016). Small.

[cit21] Kim D.-J., Shin H.-I., Ko E.-H., Kim K.-H., Kim T.-W., Kim H.-K. (2016). Sci. Rep..

[cit22] Finn D. J., Lotya M., Coleman J. N. (2015). ACS Appl. Mater. Interfaces.

[cit23] Li G. , Roberts R. C. and Tien N. C., The 13th IEEE Sensors Conference (SENSORS 2014), IEEE, Valencia, 2014, p. 1687

[cit24] Gupta S., Li G. J., Roberts R. C., Jiang L. J. (2014). Electron. Lett..

[cit25] Cervantes S. D. A., Pagan A., Santesteban B. M., Cenis J. L. (2019). Sci. Rep..

[cit26] Asakura T., Endo M., Tasei Y., Ohkudo T., Hiraoki T. (2017). J. Mater. Chem. B.

[cit27] Zhu Y., Sun W., Luo J., Chen W., Cao T., Zheng L., Dong J., Zhang J., Han Y., Chen C., Peng Q., Wang D., Li Y. (2018). Nat. Commun..

[cit28] Wang S.-D., Zhang K.-Q. (2016). Mater. Lett..

[cit29] Zhu B., Wang H., Leow W. R., Cai Y., Loh X. J., Han M.-Y., Chen X. (2016). Adv. Mater..

[cit30] Sencadas V., Garvey C., Mudie S., Kirkensgaard J. J. K., Gouadec G., Hauser S. (2019). Nano Energy.

[cit31] Farokhi M., Mottaghitalab F., Reis R. L., Ramakrishna S., Kundu S. C. (2020). J. Controlled Release.

[cit32] Wang S.-D., Ma Q., Wang K., Chen H.-W. (2018). ACS Omega.

[cit33] Huang L., Li C., Yuan W., Shi G. (2013). Nanoscale.

[cit34] Wen D.-L., Liu X., Deng H.-T., Sun D.-H., Qian H.-Y., Brugger J., Zhang X.-S. (2019). Nano Energy.

[cit35] Santos M. V., Santos S. N. C., Martins R. J., Almeida J. M. P., Paula K. T., Almeida G. F. B., Ribeiro S. J. L., Mendonça C. R. (2019). J. Mater. Sci.: Mater. Electron..

[cit36] Chen J., Xin W., Kong X.-Y., Qian Y., Zhao X., Chen W., Sun Y., Wu Y., Jiang L., Wen L. (2020). ACS Energy Lett..

[cit37] Rockwood D. N., Preda R. C., Yücel T., Wang X., Lovett M. L., Kaplan D. L. (2011). Nat. Protoc..

[cit38] Wang H., Zhu B., Wang H., Ma X., Hao Y., Chen X. (2016). Small.

[cit39] Malinowski C., He F., Zhao Y., Chang I., Hatchett D. W., Zhai S., Zhao H. (2019). RSC Adv..

[cit40] Liu Y., Bao R., Tao J., Li J., Dong M., Pan C. (2020). Science Bulletin.

[cit41] Min K., Umar M., Seo H., Yim J. H., Kam D. G., Jeon H., Lee S., Kim S. (2017). RSC Adv..

[cit42] Jayathilake D. S. Y., Sagu J. S., Wijayantha K. G. U. (2019). Mater. Lett..

[cit43] Suganuma Y., Sasaki M., Nakayama T., Muroyama M., Nonomura Y. (2017). Proceedings.

[cit44] Allard L. F., Bigelow W. C., Wu Z., Overbury S. H., Unocic K. A., Chi M., Carpenter W. B., Walden F. S., Thomas R. L., Gardiner D. S., Jacobs B. W., Nackashi D. P., Damiano J. (2015). Microsc. Microanal..

[cit45] Lin S.-Y., Zhang T.-Y., Lu Q., Wang D.-Y., Yang Y., Wu X.-M., Ren T.-L. (2017). RSC Adv..

[cit46] Lee D., Bang G., Byun M., Choi D. (2020). Thin Solid Films.

[cit47] Li M., Minoura N., Dai L., Zhang L. (2001). Macromol. Mater. Eng..

[cit48] Nair K. G., Jayaseelanand D., Biji P. (2015). RSC Adv..

[cit49] Sorel S., Bellet D., Coleman J. N. (2014). ACS Nano.

