# Supplementary material for: Highly transparent and flexible Ag nanowire-embedded silk fibroin electrodes for biocompatible flexible and transparent heater
Source: RSC Adv. 2020 Aug 28;10(53):31856–62. doi: 10.1039/d0ra05990k (PMC9056558; doi:10.1039/d0ra05990k)
Supplement: RA-010-D0RA05990K-s001 [file RA-010-D0RA05990K-s001.pdf]

Electronic Supplementary Material (ESI) for RSC Advances.
This journal is © The Royal Society of Chemistry 2020

Supporting Information

Highly Transparent and Flexible Ag Nanowire-Embedded Silk Fibroin substrates for Flexible, Transparent, and Biocompatible Heaters

Jin-Hyeok Park[a], Hae-Jun Seok[a], Eswaran Kamaraj[b], Sanghyuk Park*[b], and Han-Ki Kim*[a]

[a]School of Advanced Materials Science and Engineering, Sungkyunkwan University, 2066, Seobu-ro, Jangan-gu, Suwon-si, Gyeonggi-do 16419, Republic of Korea.
[b]Department of Chemistry, Kongju National University, 56, Gongjudaehak-ro, Gongju-si, Chungcheongnam-do 32588, Republic of Korea.

[a] E-mail: hankikim@skku.edu Fax: +82-31-201-2462; Tel: +82-31-205-2462

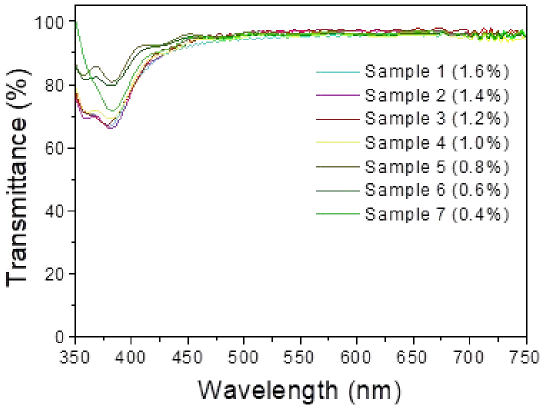

Fig. S1 The optical transmittance of Ag NWs on SF films which is measured at SF substrate background as a function of weight percentages of Ag NWs dispersed in isopropanol solvent .

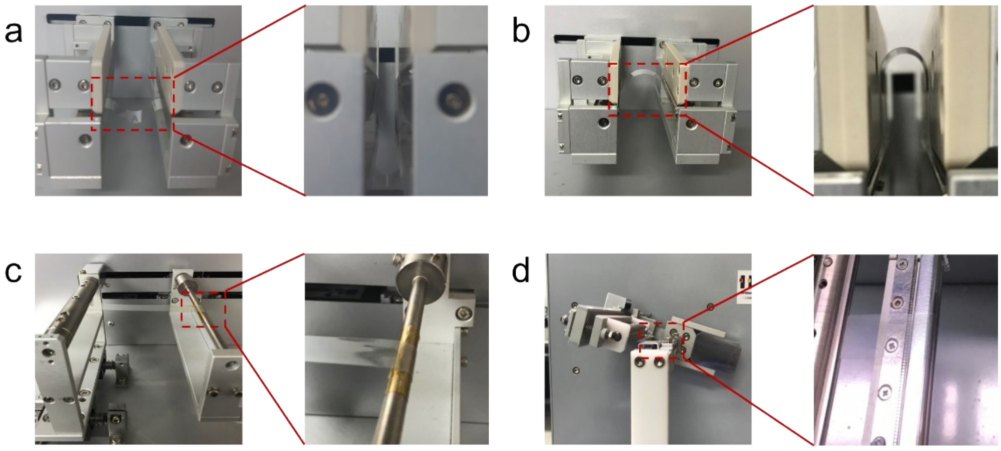

Fig. S2 Photography of laboratory designed (a) inner bending, (b) outer bending, (c) rolling, and (d) folding test systems.

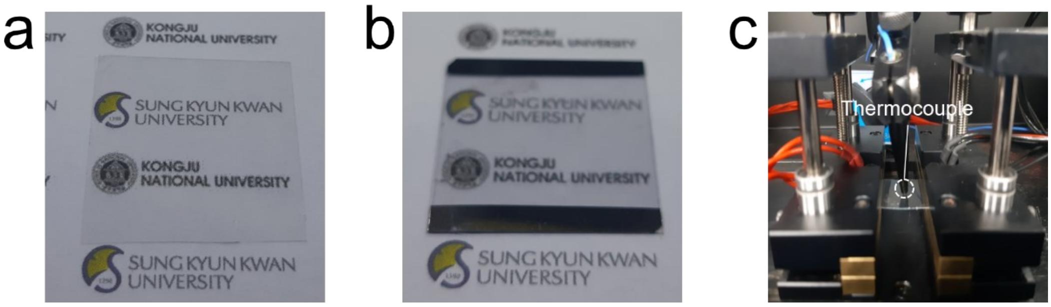

Fig. S3 The pictures of process sequence for Ag NWs/SF based TFH fabrication (a) preparation of Ag NWs/SF electrode films with a size of 2.5×2.5 cm^2, (b) formation of contact metal (silver) by DC magnetron sputtering method, and (c) connection of contact metal area to DC power supply and measurement of temperature using thermocouple mounted onto the Ag NWs/SF based TFHs.

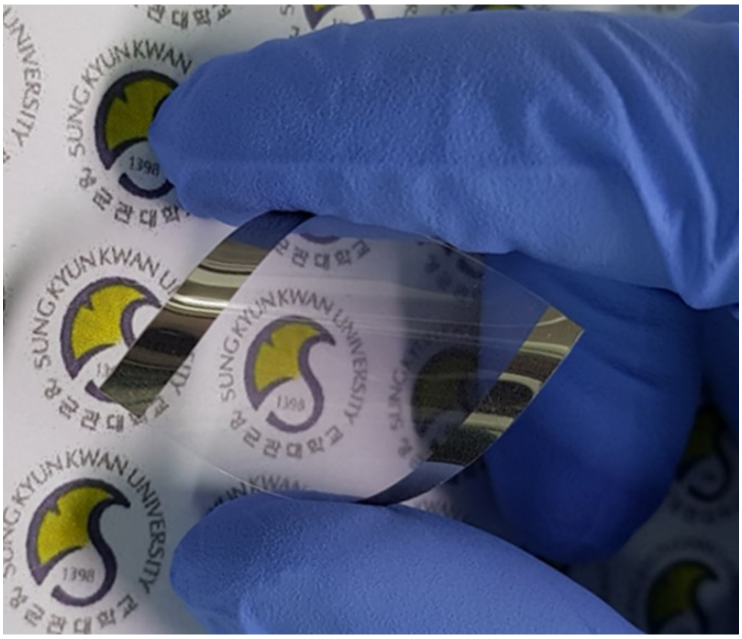

Fig. S4 Photograph of transparent and flexible Ag NWs/SF based TFH device.

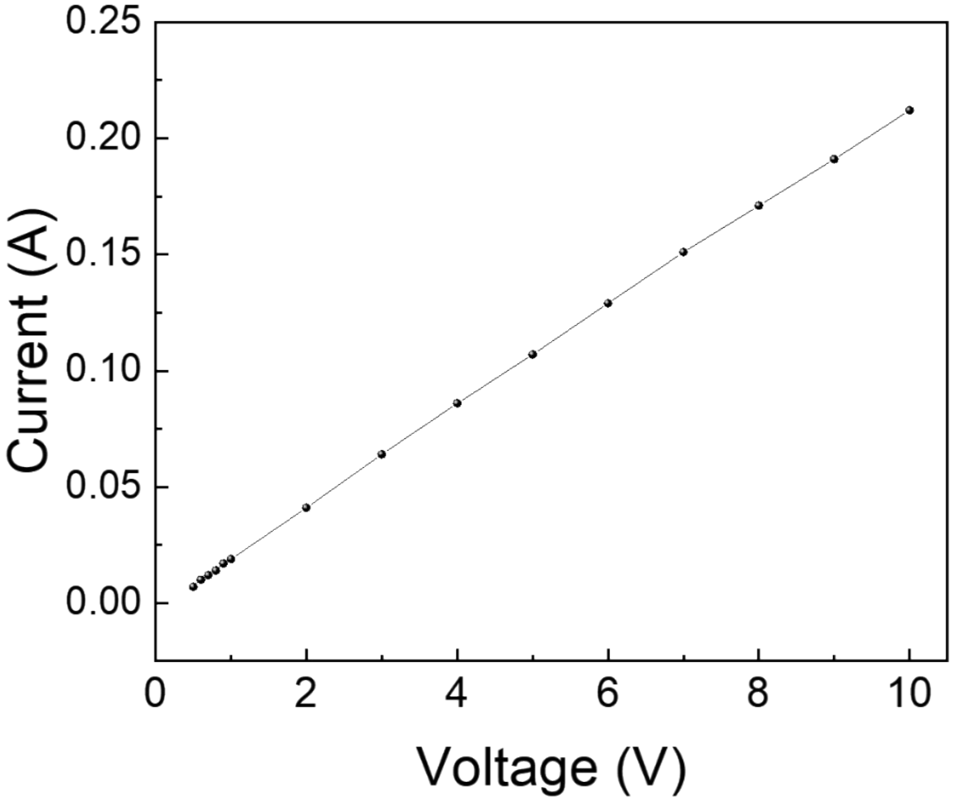

Fig. S5 I-V characteristics of Ag NW-embedded SF-based TFH *in-situ* measured with temperature profile.

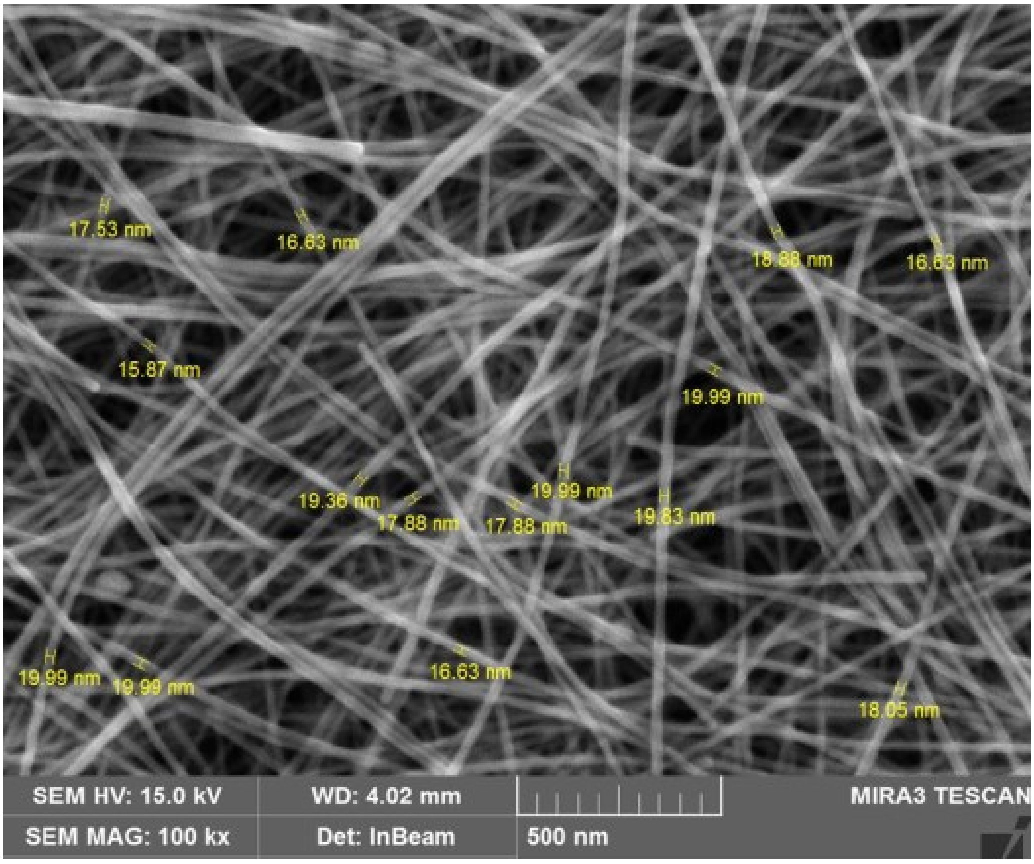

Fig. S6 SEM images of prepared Ag nanowires for the transparent electrode.

	**Sample 1** (1.6% AgNWs)	**Sample 2** (1.4% AgNWs)	**Sample 3** (1.2% AgNWs)	**Sample 4** (1.0% AgNWs)	**Sample 5** (0.8% AgNWs)	**Sample 6** (0.6% AgNWs)	**Sample 7** (0.4% AgNWs)
**AgNWs/PET**	18.4(±1.6)	16.7(±1.6)	15.8(±1.3)	22.4(±1.9)	38.8(±4.8)	62.8(±6.7)	95.0(±6.6)
**AgNWs/SF**	18.1(±1.5)	16.3(±1.5)	15.5(±1.3)	22.4(±1.4)	36.9(±3.4)	62.5(±9.7)	93.6(±8.3)

Table S1 The sheet resistance of Ag NWs on PET film and SF film as a function of weight percentages of Ag NWs dispersed in isopropanol solvent.
